# Neural tracking of prosodic and statistical rhythms jointly supports artificial language learning

**DOI:** 10.1016/j.isci.2026.116458

**Published:** 2026-06-24

**Authors:** Lorenzo Titone, Burkhard Maess, Lars Meyer

**Affiliations:** 1 Max Planck Research Group Language Cycles, Max Planck Institute for Human Cognitive and Brain Sciences, Leipzig, Germany; 2 Methods and Development Group Brain Networks, Max Planck Institute for Human Cognitive and Brain Sciences, Leipzig, Germany; 3 Department of English and Linguistics, Johannes Gutenberg University Mainz, Mainz, Germany; 4 Clinic for Phoniatrics and Pedaudiology, University Hospital Münster, Münster, Germany

**Keywords:** medical imaging, cognitive neuroscience, biocomputational method, high-performance computing in bioinformatics

## Abstract

Neural tracking of prosodic and statistical patterns supports speech segmentation and word learning. Yet, acoustic and abstract patterns have proven difficult to dissociate empirically. Our study employs an artificial language learning paradigm generated with a method that orthogonalizes acoustic and statistical patterns in auditory stimuli. MEG recordings from 32 human adults who had been exposed to continuous syllable streams reveal distinct brain sources tracking periodic acoustic, prosodic, and statistical patterns. After encoding, M200 and M400 field responses to isolated trisyllabic chunks show that participants retained both prosodic and statistical patterns for learning pseudowords from the syllable streams. Behavioral data extend our findings, suggesting not only that integrating both acoustic and abstract patterns is critical to learning, but also that prior knowledge of the pseudowords is sufficient for recognition. The findings advance our understanding of the neural sources that encode and integrate acoustic and statistical patterns during and after language learning.

## Highlights

• Prosodic and statistical patterns support artificial language learning
• Distinct brain areas synchronize to prosodic and statistical patterns
• M200 and M400 fields dissociate learning of prosodic and statistical patterns
• Frontal areas reduce synchronization to irrelevant acoustic stimulus dimensions

## Graphical abstract

**Figure:**
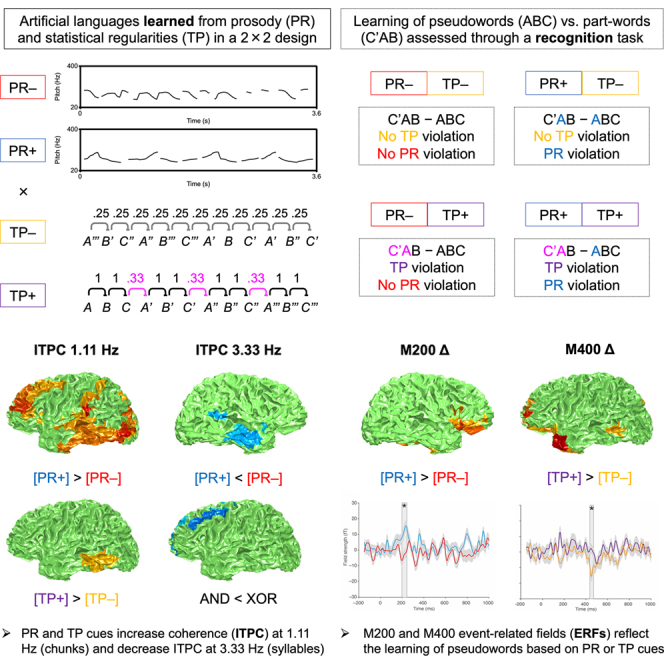

## Introduction

Speech is a complex signal rich in acoustic and statistical patterns. Our brains leverage both types of patterns to segment speech and infer its abstract structures.^1^^,^^2^ Acoustic patterns, such as amplitude or pitch modulations, enable a metrical segmentation strategy for word extraction.^3^^,^^4^ In parallel, statistical patterns, such as higher transitional probabilities (TPs) between syllables that form words,^5^ offer non-acoustic cues that inform word boundaries and support pseudoword learning.^6^ Speech prosody crosses the boundary between acoustic and abstract patterns, since it provides acoustic cues that support linguistic structure building.^7^^,^^8^ Accordingly, behavioral studies found that coherent prosodic and statistical patterns in speech boost pseudoword learning^9^^,^^10^ and their mapping to objects.^11^ Yet, the neural circuits underlying the integration of both patterns are unknown.

Frequency-tagging designs^12^ are used to dissociate the tracking of rhythmic stimuli features.^13^ For instance, frequency-tagging studies found neural tracking at the rate of both acoustic and non-acoustic patterns in isochronous speech sequences, such as acoustic (e.g., syllables) or abstract (e.g., words) units,^14^ or statistical patterns identifying artificial pseudowords.^15^ Furthermore, previous EEG studies found that prosodic and statistical patterns elicit distinct evoked brain responses. In particular, Cunillera et al.^16^ found a P200 difference in response to differently stressed syllable sequences, on top of an N400 difference reflecting an online marker of speech segmentation during the learning of TP patterns. In addition, Elmer et al.^17^ found a reconfiguration of these neural responses during the learning process. Based on these results, prosody and TP patterns appear to modulate the P200 and N400 responses, respectively.^16^^,^^17^

The literature suggests that both prosody and statistical patterns support pseudoword learning. However, the orthogonal experimental manipulation of acoustic and statistical patterns in the prior literature may not always have been successful in controlling for confounding types of stimulus rhythmicity. In fact, the neural encoding of phonological and acoustic patterns^18^^,^^19^ may have confounded the neural tracking of statistical patterns in prior work,^20^^,^^21^ limiting scientific interpretability. As further confounds, linguistic entrenchment with a known language^22^ and phonotactics^23^ are likely to impact the learning of another (artificial) language. To overcome these limitations, we developed a toolbox to achieve *ad-hoc* control of such rhythmicity and linguistic confounds in artificial speech.^24^

**Figure 1:**
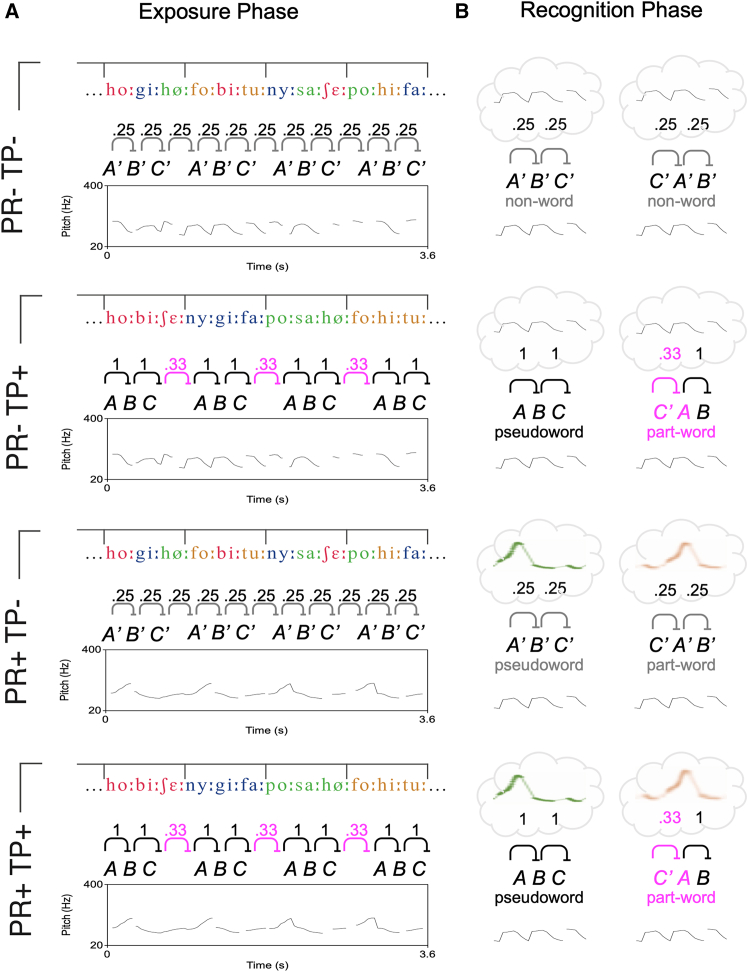
Experimental design (A) Exposure phase. The panels depict the manipulation of prosodic (PR) and transitional probability (TP) patterns between syllables across the four conditions. The prosodic pattern in PR + conditions marked trisyllabic prosodic boundaries via a rise in pitch, which replaced the original pitch of the artificially synthesized syllables (in PR− conditions). Parallel to the manipulation of prosody, syllables were arranged either in a TP-structured stream (TP+) or in a TP-uniform stream (TP−). In TP + streams, triplets of syllables cluster together to form pseudowords, which are color-coded in the syllable sequences (top). Across pseudoword boundaries, TPs drop from 1 to 0.33 (magenta) in TP+, but not in TP− conditions (flat TPs of 0.25). The vertical lines above each syllable sequence point to the fixed locations in the streams where PR or TP boundaries could repeat periodically every three syllables. (B) Recognition phase. The panel displays the two types of syllable sequences (i.e., ABC pseudowords vs. C’AB part-words) presented during the forced-choice task that directly followed the exposure phase in each condition. All sequences were played with no pitch manipulation (bottom). The elements in the clouds reflect the supposed memory of PR and TP patterns from the immediate prior exposure. Notably, pseudowords and part-words should be indistinguishable (non-words) after exposure to PR– TP− streams. Two distinct lexicons were assigned to either PR condition (counterbalanced across participants), and the order of presentation of the conditions was balanced across participants according to a Latin square design (Table S1).

In this study, we combined magnetoencephalography (MEG) with frequency-tagging stimuli. We aimed to dissociate the neural circuits that underlie the tracking and integration of prosodic and statistical patterns during artificial language learning. To that end, we exposed human adults (*N* = 32) to isochronous syllable streams at 3.33 Hz, wherein prosodic (PR) and statistical (TP) cues delineate trisyllabic chunks at 1.11 Hz (Figure 1A). After the exposure phase to each individual condition, participants performed a two-alternative forced-choice recognition task and selected the most familiar of two chunks that could either match or violate PR or TP patterns from the prior exposure (Figure 1B).

## Results

We find distinct MEG sources associated with the learning and recognition of PR and TP patterns. We first dissociate the brain sources that afford acoustic tracking at the rate of syllables (3.33 Hz), and—orthogonally—neural tracking at the rate of prosodic (PR) and statistical (TP) patterns (1.11 Hz) using power spectral density and inter-trial phase coherence (ITPC) analyses of the MEG activity during the exposure to the artificial speech streams. Subsequently, we find a double dissociation of the M200 and M400 event-related field (ERF) responses to valid or invalid pseudowords during the recognition phase, depending on the preceding exposure to PR and TP patterns, respectively. Behavioral analyses further suggest that PR and TP patterns are both needed for learning.

**Figure 2:**
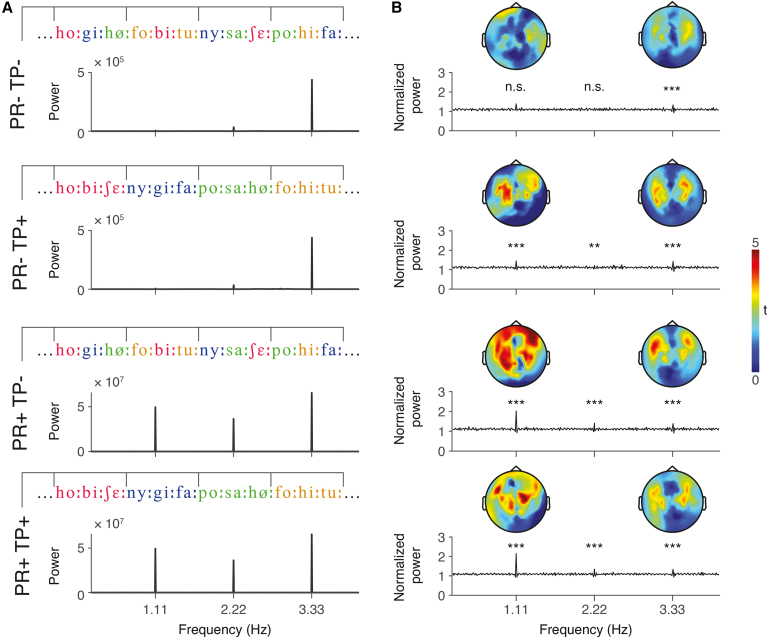
Frequency-tagging and power spectral density results (A) Amplitude modulation spectra in each condition display clear acoustic peaks at the syllable rate (3.33 Hz) across conditions, and at the rate of prosody (1.11 Hz) and its harmonic (2.22 Hz) in PR + conditions. In contrast, only a small peak at 2.22 Hz is visible in the PR– conditions. (B) Grand-averaged normalized power spectra and sensor-level t maps at the syllable (3.33 Hz) and chunk (1.11 Hz) rates in the four conditions. Stars indicate significance of cluster-based permutation tests within condition (∗∗, *p* < 0.01; ∗∗∗, *p* < 0.001).

We used a 2 × 2 frequency-tagging paradigm to dissociate PR and TP patterns. The latter shaped a pseudoword rate at 1.11 Hz within isochronous streams of syllables at 3.33 Hz. In PR− streams, the amplitude modulation spectra of the syllable streams (Figure 2A) show a large acoustic peak at the rate of syllables (3.33 Hz), but no clear peak at the rate of chunks (1.11 Hz), or its harmonic (2.22 Hz). In contrast, all such peaks are larger in PR + streams. Acoustic peaks at the pseudoword rate may vary across lexicons due to acoustic strength differences across phonemes that appear at regular positions.^20^ However, we observe only small acoustic peaks at the pseudoword rate, likely resulting from the positional controls of our stimuli in terms of manner of articulation and vowel identity, which might otherwise lead to distinctive spectral characteristics of the sound. While acoustic regularities cannot be fully eradicated within a stream, they can be factored out using a position-controlled TP-uniform baseline,^20^ which allows ascribing neural tracking effects at the syllable and chunk rates to the presence of PR or TP patterns in the streams. In addition, we designed our stimuli against the further confounding of phonological feature rhythmicity and statistical regularities at 1.11 Hz.^20^^,^^24^

After learning, we measured neural and behavioral responses to pseudowords and deviants. Each trial (*n* = 48 per condition) presented one pseudoword and one part-word sequentially (Table S2). Here, pseudowords and part-words could be distinguished based on the PR or TP patterns that were present in the prior exposure (Figure 1B). PR + streams, but not PR−, carried an acoustic marking at 1.11 Hz, locked on every first syllable, which is absent in the recognition phase. Therefore, pseudowords, with an ABC structure, present a marked pitch violation at the onset syllable, A, after PR + streams; whereas part-words, with a C'AB structure, present a weaker violation, as it stands at the second syllable position. In contrast, neither pseudowords nor part-words carry pitch violations after PR− streams. Orthogonal to the PR contrast, we manipulated statistical regularities (TPs) between syllables. After TP + streams, pseudowords presented TPs of 1 both between A and B, and between B and C; but part-words presented TPs of 0.33 between C′ and A. In contrast, in TP− streams, TPs were equal (0.25) across syllables (A′B′C′) and do not mark pseudowords. Thus, pseudowords and part-words presented PR or TP differences within each contrast that surpass systematic differences in the first syllable (A or C) and phonotactics.

### PR and TP patterns modulate neural tracking of syllables and chunks

To quantify neural tracking, we computed the normalized power spectral density of the MEG signal in the delta band (0.2–4 Hz). Normalized power was calculated as the ratio between the observed power at each frequency in the delta band and the baseline power, averaged across the two corresponding neighboring frequencies (Figure 2B). Cluster-based permutation tests^25^ contrasting the observed and the baseline power in each condition revealed significant peaks at the syllable rate (3.33 Hz) in all conditions (all *p* < 0.001), and at the chunk rate (1.11 Hz) in both PR+ and TP + conditions (all *p* < 0.001), but not in the PR− TP− condition (*p* = 0.97). The spatial distributions of these peaks suggest that tracking of prosodic and statistical patterns at the same rate can be dissociated spatially.

Our power spectral density results show that the brain tracks acoustic, PR, and TP patterns. Based on this finding, we analyzed the ITPC at the syllable (3.33 Hz) and chunk (1.11 Hz) rates. We modeled the main effects of PR and TP as ITPC differences in PR + relative to PR− and in TP + relative to TP− streams, respectively; and the interaction of PR × TP as the ITPC difference of differences in TP + versus TP− streams within PR + relative to PR− streams. We performed cluster-based permutation tests to assess whether ITPC at 1.11 Hz increased, while ITPC at 3.33 Hz decreased, due to the presence versus absence of PR or TP patterns. We used dynamic imaging of coherent sources^26^ to reconstruct the brain sources of PR and TP effects on ITPC at the two rates. Source-level ITPC was averaged within each of the 360 regions of the HCP-MMP1 atlas^27^ and critical contrasts were tested using separate one-sided paired sample t-tests (Table S3). Only 25 out of 32 participants contributed to all of our source-level analyses due to lack of MRI scans (five) or co-registration failure (two).

**Figure 3:**
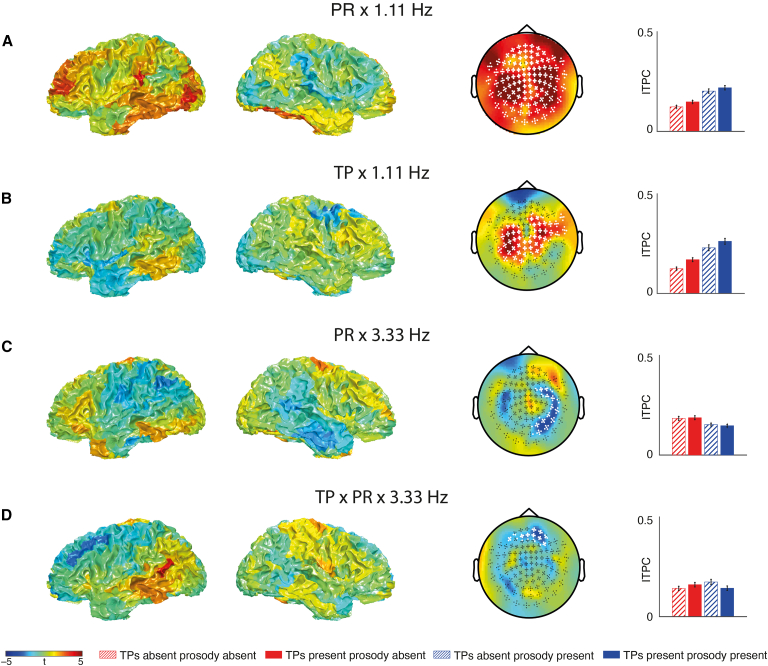
ITPC results from the exposure phase Each panel displays, from left to right: (i) source-level t maps; (ii) sensor-level t maps, showing the clusters of sensors displaying significant ITPC differences in each contrast (white dots) resulting from cluster-based permutation tests (*p* < 0.05); (iii) grand-averaged ITPC within the respective sensor-level cluster. Data represent mean ± standard error of the mean (SEM) in each of the four conditions across participants. (A) PR × 1.11 Hz effect. (B) TP × 1.11 Hz effect. (C) PR × 3.33 Hz effect. (D) PR × TP × 3.33 Hz effect.

We found significant differences in ITPC at the rates of interest due to PR or TP patterns (Figure 3). Sensor-level cluster-based permutation tests show that ITPC at 1.11 Hz increased when PR patterns were present (Figure 3A, mass-cluster t [31] = 2003.7, *p* < 0.001). This PR effect is source-localized to left fronto-temporal areas, peaking in the left rostral frontal operculum (t [24] = 3.55, uncorrected *p* = 0.0008). ITPC at 1.11 Hz also increased when TP patterns were present (Figure 3B, mass-cluster t [31] = 713.33, *p* < 0.001). This TP effect source-localized to left temporal areas, peaking in the left area PHT (t [24] = 1.78, uncorrected *p* = 0.0438). In contrast, ITPC at 3.33 Hz decreased when PR patterns were present (Figure 3C, mass-cluster t [31] = −174.43, *p* < 0.05). This PR effect is source-localized to right superior temporal areas, peaking in the right superior temporal sulcus (t [24] = −2.34, uncorrected *p* = 0.0138). Furthermore, ITPC at 3.33 Hz was modulated by a PR × TP effect (Figure 3D, mass-cluster t [31] = −123.71 *p* < 0.05). This interaction effect was source-localized to prefrontal areas, peaking in the left area 46 (t [24] = −2.53, uncorrected *p* = 0.0092). No differences were found in any other contrast (all *p* > 0.22). Post-hoc analyses resolved the interaction as decreased ITPC in PR− TP− versus PR− TP+ (t [31] = −1.99, *p* = 0.05) and PR + TP− (t [31] = −2.96, *p* < 0.01) streams, but increased ITPC in PR + TP− versus PR + TP+ (t [31] = 2.77, *p* < 0.01) streams. In sum, syllable tracking decreased when PR and TP patterns jointly identified structured or uniform streams, but it increased when PR or TP patterns were present alone.

Based on our overall ITPC findings, we investigated how ITPC at 1.11 Hz evolved in time. In particular, we found two clusters that displayed increased tracking of PR (Figure 3A) or TP (Figure 3B) patterns at the chunk rate (1.11 Hz). Assuming bottom-up and top-down processing of PR and TP patterns, respectively, we then expected ITPC in these two clusters to be differently affected by the elapsed time of exposure. We computed a moving average of ITPC within each sensor-level cluster from consecutive overlapping epochs of 50 chunks (Figure S1). To estimate the effect of time on ITPC, we designed separate linear mixed-effects models^28^ for the two-halves of exposure. In PR + streams, ITPC increased during the first half of exposure (t [4543] = 3.58, *p* < 0.001) and decreased during the second half (t [4543] = −4.02, *p* < 0.001). Instead, in TP + streams, ITPC increased with time only during the first half of exposure (t [4543] = 8.45, *p* < 0.001), but not in the second half (t [4543] = −1.78, *p* = 0.07). These findings indicate a slow adaptation to PR patterns during the second half of the exposure, and a faster neural tracking of pseudowords, as TP patterns accrued within the first 3 min of the exposure.

### M200 and M400 fields are sensitive to violations of PR or TP patterns

After the exposure to each condition, we measured ERF responses to pseudowords and part-words. We hypothesized a dissociation of prosodic and word-form processing, reflecting the encoding of PR and TP patterns. Respectively, we predicted differences in the M200 and M400 responses to pseudowords and part-words that respect or violate PR and TP patterns from the prior exposure. In line with prior studies,^29^^,^^30^^,^^31^^,^^32^ we first identified the latencies of interest for these two ERFs based on the full-width half-max (FWHM) of the most prominent peaks in the grand-average root-mean-square signal across all trials (Figure S2). We then computed the difference between part-words and pseudowords by condition. To dissociate the PR and TP effects on the M200 and M400 responses, we modeled the PR × M200 effect as the M200 difference after PR + versus PR− streams, and the TP × M400 effect as the M400 difference after TP + versus TP− streams. Likewise, we modeled the PR × M400 and the TP × M200 effects. We tested the four contrasts with cluster-based permutation tests at the sensor level. Next, we source-reconstructed the ERF responses using linear-constrained minimum-variance beamforming^33^ and assessed the significant contrasts in each atlas region with separate paired t-tests (Table S4).

**Figure 4:**
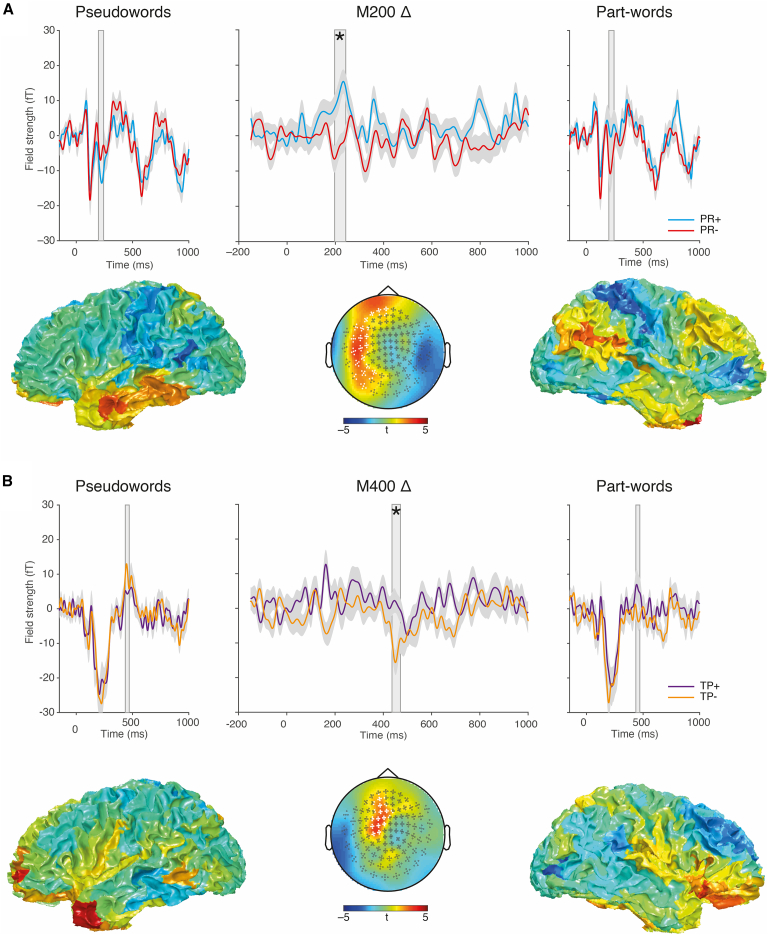
ERF results from the recognition phase The top panels display the grand-averaged ERF traces for pseudowords (left) and part-words (right), or their difference (part-word – pseudoword) in the respective sensor-level clusters for each contrast. The difference between Gray areas indicates the latencies of interest (196–242 ms; 435–470 ms) based on the FWHM of grand average RMS peaks (Figure S2). Solid lines and shaded areas indicate the grand-averaged time course and SEM within the respective sensor-level cluster. The bottom panels present sensor-level t maps (middle), showing the clusters of sensors displaying significant ERF differences within the respective contrast (white dots); and source-level t maps in the left and right hemispheres. Stars indicate significance of cluster-based permutation tests (*p* < 0.05). (A) PR violations influence the M200. (B) TP violations influence the M400.

We found two sensor-level clusters supporting a dissociation of PR and TP on the ERF responses. In particular, we found a significant PR × M200 effect (Figure 4A; mass-cluster t [31] = 221.88, *p* < 0.05), and a significant TP × M400 effect (Figure 4B; mass-cluster t [31] = 172.12, *p* < 0.05), whereas the PR × M400 and TP × M200 were not significant (all *p* > 0.09). Post-hoc tests revealed that M200 responses to part-words increased after PR + versus PR− streams (t [31] = 3.03, *p* < 0.01), while M200 responses to pseudowords relative to part-words decreased after PR + streams (t [31] = −3.78; *p* < 0.001). In contrast, M400 responses to part-words increased after TP + versus TP− streams (t [31] = 2.25, *p* < 0.05), while M400 responses to pseudowords relative to part-words increased after TP− streams (t [31] = 3.75, *p* < 0.001). Source-level analyses localized the PR × M200 effect to right inferior frontal areas and left temporal areas, peaking in the right area 47s (t [24] = 3.00, uncorrected *p* = 0.0031); and the TP × M400 effect to left anterior temporal and anterior frontal areas, peaking in the left dorsal temporal gyrus (t [24] = 4.21, uncorrected *p* = 0.0001). Overall, these results dissociate the timing and the sources of ERF differences in response to syllable sequences that violate the PR or TP patterns (part-words) or not (pseudowords).

Control analyses tested the M200 and M400 effects of PR and TP in larger time windows. We first extended the narrow data-driven range of latency (FWHM) to an arbitrary ±50 ms window around the peak, as well as to an average across the whole *a priori* window in each contrast (see also Method Details). We find that the M200 effect survived when tested on both these time windows (i.e., 170–270 ms: mass-cluster t (31) = 192.72, *p* < 0.05; 170–250 ms: mass-cluster t (31) = 234.60, *p* < 0.05). In contrast, the M400 effect did not survive in either of the two windows (i.e., 403–503 ms: *p* = 0.14; 300–500 ms: *p* = 0.28). Moreover, no cluster was found to support significant ERF differences in either contrast when testing the whole post-stimulus (i.e., 0–1000 ms) window (without averaging over time). In total, the PR × M200 effect survived to a larger window around the peak, while the TP × M400 effect was significant only in a narrower window.

### PR and TP patterns are both needed for learning pseudowords

**Figure 5:**
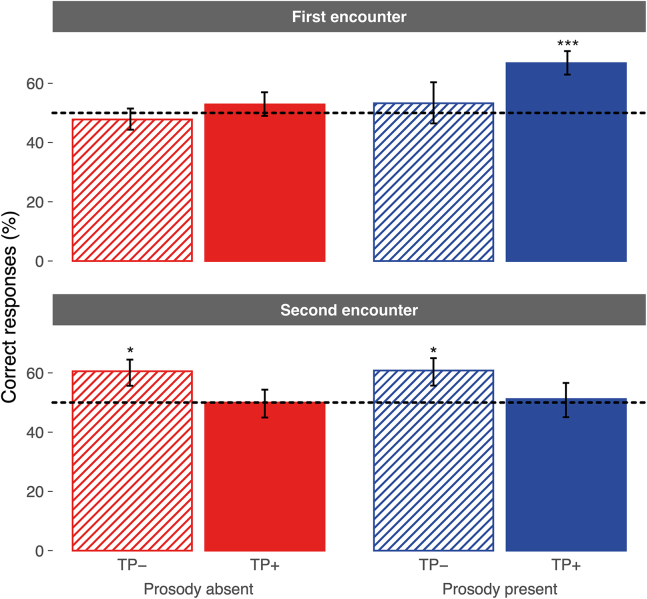
Behavioral results The bar graphs display the overall percentage of pseudoword recognition in the task as a function of condition (PR and TP patterns) and priors in the task (first and second encounters). By design, four groups of participants had different condition orders (Table S1), for which participants were tested twice on the same task after being exposed to the same lexicon (same PR condition) in either the TP condition. Stars indicate above-chance (50%, dotted line) significance of one-sample t-tests within group (∗, *p* < 0.05; ∗∗∗, *p* < 0.001). Data are represented as mean ± SEM.

Participants’ behavioral accuracy in the task was above chance after PR and TP were both present. One-sample t-tests showed above-chance (>50%) recognition of pseudowords as more familiar than part-words only in the PR + TP + condition (t [31] = 2.40, *p* < 0.05; all other *p* > 0.05). Yet, since the same recognition task was administered twice in every session—i.e., after both TP+ and TP− streams (Tables S1 and S2)—we considered carry-over effects due to experimental history (HI). We thus split our dataset post-hoc to code an HI− portion of data when participants encountered the lexicon and task for the first time, and an HI + condition, when participants encountered the same lexicon for the second time in the same PR condition. We assessed the main effects and interactions of PR, TP, and HI on single-trial recognition accuracy using binomial mixed-effects models. A baseline model with only a random intercept per participant showed a worse fit than an extended model including the PR × TP × HI interaction (χ2 [7] = 95.13, *p* < 0.001). The best-fit model exposed a TP × HI effect (z = −2.39, *p* < 0.05) and a main effect of HI (z = 3.43, *p* < 0.001). Post-hoc tests showed that performance improved not only after PR+ (t [60] = 2.37, *p* < 0.05) and TP + streams (t [60] = 2.27, *p* < 0.05) on the first task encounter (HI−), but also after TP− streams (t [60] = −2.37, *p* < 0.05) on the second encounter (HI+). One-sample t-tests showed above-chance performance only in the PR + TP + condition within HI− (t [15] = 4.23, *p* < 0.001), and in the PR− TP− (t [15] = 2.36, *p* < 0.05) and PR + TP− conditions (t [15] = 2.31, *p* < 0.05) within HI+ (all other *p* > 0.24). These results suggest that both PR and TP patterns are needed for learning previously unheard artificial pseudowords, but also that previously known pseudowords are still remembered later on, that is, after an intervening TP− stream (Figure 5).

## Discussion

Our MEG study dissociates the neural sources encoding prosodic and statistical speech rhythms during artificial language learning. Participants listened to artificial languages embedded within frequency-tagged speech. Spectral analyses of the exposure phase to the syllable streams show that the presence of PR or TP patterns modulated the ITPC at 1.11 Hz or 3.33 Hz in distinct MEG-source reconstructed brain areas. Temporal and frontal regions show differences in neural tracking at the syllable and chunk rates based on the presence of either or both PR and TP patterns. Then, the encoding of PR and TP patterns led to distinct M200 and M400 field differences between pseudoword and part-words during the offline recognition task that followed each exposure phase. Behaviorally, linear mixed-effects models suggest that PR and TP patterns are both needed for discovering the pseudowords after their first presentation, while recognition was also affected by a carry-over effect due to the prior history of the experimental session.

### Distinct neural circuits track syllables and chunks based on PR and TP patterns

Prosodic (PR) and statistical (TP) patterns modulate chunk (1.11 Hz) and syllable (3.33 Hz) tracking (Figure 2). Previous statistical learning studies have found increased neural tracking of pseudowords over syllables in TP + relative to TP− streams, by analyzing a word learning index, defined as the ratio of chunk-rate and syllable-rate ITPC.^34^^,^^35^^,^^36^^,^^37^^,^^38^ Expanding on this evidence, our findings dissociate rate-specific PR or TP effects on ITPC and localize these effects in distinct brain sources. Neural tracking of PR patterns at 1.11 Hz (Figure 3A) source-localized to left fronto-temporal regions within the language network,^39^ which are involved in processing lexicalized prosody.^40^ Instead, neural tracking of abstract TP patterns at 1.11 Hz (Figure 3B) source-localized to left posterior temporal regions that are structurally connected to the arcuate fasciculus,^39^ which mediates word learning.^41^ This TP effect might reflect the abstract tracking of statistical regularities, beyond the acoustics, which may interact with the encoding of pseudowords as individual elements.^34^^,^^42^ Furthermore, PR patterns led to decreased tracking at 3.33 Hz source-localized to the right superior temporal gyrus (Figure 3C), which is known to be involved in tracking syllables^43^ and prosody.^44^^,^^45^

In addition to the main effects, PR and TP jointly reduce syllable tracking. We found a PR × TP interaction in a prefrontal cluster, displaying reduced tracking at 3.33 Hz when PR and TP patterns were either both present or both absent. This effect is source-localized to Area 46, in the left dorsolateral prefrontal cortex. This brain region is involved in the integration of prosody across parts of the language network, processing acoustic and abstract patterns.^46^ More generally, the prefrontal cortex is thought to orient cognitive resources to process stimuli that carry high informational value.^47^ The observed PR × TP effect is in line with both of these accounts. When both PR and TP patterns are present, syllable tracking might be reduced because the first syllable of a pseudoword contains all the informational value. Instead, when both patterns are absent, syllable tracking might be reduced because no acoustic or abstract structures are there to be found. In contrast, when only one type of pattern is present, syllable tracking might increase to promote an acoustic search for candidate patterns in the syllable stream.

### M200 and M400 fields suggest encoding of PR and TP patterns

In the recognition phase, participants heard *ABC* pseudowords and *C’AB* part-words. We predicted different evoked responses to these items based on the prior exposure to PR or TP patterns. As PR patterns always stressed the onset syllable of each pseudoword, participants could have formed a prosodic expectation of finding a stressed syllable *A* at pseudoword onset. This expectation would lead to a stronger omission response for *ABC* over *C’AB* chunks devoid of prosody after PR + but not after PR− streams. In line with this idea, we observed increased M200 responses to pseudowords than part-words in the PR contrast, suggesting a neural sensitivity to an auditory memory that mingled with statistical learning.^48^^,^^49^^,^^50^ This sensitivity to violations of previous PR patterns in the recognition phase would also support a prosodic bootstrapping account,^9^^,^^17^ although the term has a broader meaning in the language acquisition literature.^51^ Beyond PR, different statistical patterns characterized the two test chunks in the TP contrast. This difference led to reduced M400 responses to pseudowords and increased M400 responses to part-words, after TP + relative to TP− streams. Notably, while prior work observed a larger N400 for pseudowords during the learning process,^16^^,^^17^^,^^34^^,^^48^^,^^52^ our results in the post-learning phase show an M400 effect that goes in the opposite direction. However, it is important to note that previous work also showed reduced N400 responses to pseudowords after learning, suggesting a reconfiguration of this response.^17^ Since we measured the ERF differences after the learning has occurred, our M400 results appear consistent with a familiarity effect that emerged only after artificial word-forms had been learned and consolidated, becoming smaller when TP patterns rendered pseudowords more expected and thus easier to process.^53^

In total, we find that the M200 and M400 responses are distinctively modulated by PR and TP violations. These results expand on prior evidence that P200 and N400 differences to prosodic and statistical violations vary throughout the exposure,^16^^,^^17^ by showing a double dissociation of M200 and M400 responses after the learning of pseudowords from TP or PR patterns. Furthermore, our source-level analyses dissociate the neural generators of these effects. The PR × M200 effect was source-localized to the right inferior frontal gyrus and to left middle temporal regions, which might thus be involved in recalling pseudowords aided by the prior PR patterns. In contrast, the TP × M400 effect was source-localized to the left temporal pole, connected to the uncinate fasciculus,^39^ and functionally important for the learning of statistical dependencies.^54^

### Lexical priors, PR, and TP patterns predict pseudoword recognition

Participants recognized pseudowords over part-words above chance after PR + TP + conditions. This finding is line with previous evidence that prosody and statistical patterns are both needed to enable artificial language learning in the adult brain.^9^^,^^10^ In addition, linear mixed-effects model analyses revealed how behavioral performance was affected by the history (HI) in which conditions were presented due to our within-subject design. Strikingly, these models show that previously learned pseudowords were retained after an intervening random stream (TP−). In particular, participants who were exposed to the pseudowords in a preceding TP + stream did recognize them on the second task encounter, that is, after a TP− stream. However, those who were first exposed to a TP− stream first did not show signs of learning even after a subsequent TP + stream. Thus, prior lexical knowledge of the pseudowords, rather than task familiarity, drove the TP × HI effect. In line with recent reports,^55^^,^^56^ this finding indicates that, beyond the encoding of PR and TP patterns participants, also relied on lexical priors for recalling pseudowords. Bringing together our brain and behavioral results, we observed that the absence of TP patterns reduced neural tracking of pseudowords, but also that participants showed above-chance recognition of pseudowords following TP−, but not TP+, streams during the second task encounter (HI+). Together, these findings indicate that the presence or absence of PR and TP patterns that first enabled the learning of pseudowords (in HI−) was then disregarded, in favor of lexical knowledge, to solve the task again (in HI+). In line with previous reports,^57^ our results suggest that lexical priors were employed for correct speech segmentation, beyond low-level acoustic and statistical cues that first enabled learning. Yet, pseudowords were not learned if TP patterns emerged only at later experimental stages, further suggesting that null lexical priors inhibited the discovery of TP patterns identifying pseudowords. Therefore, both types of patterns were needed to learn previously unheard pseudowords, possibly through increased neural tracking of chunks over syllables in a fronto-temporal network. However, the recall of previously formed lexical memories was robust to the loss of statistical patterns in subsequent syllable streams.

### Limitations of the study

Our study is limited to isolated analyses of neural tracking, ERFs, and behavior. In particular, we did not investigate potential top-down control mechanisms of neural tracking with connectivity and cross-frequency coupling analyses. However, we acknowledge that the neural sources encoding syllables and pseudowords from PR and TP patterns may be part of a circuit that integrates bottom-up acoustic patterns^58^^,^^59^ with top-down lexical priors to infer abstract linguistic structure.^2^^,^^60^ Previous studies showed that top-down predictions from higher-order regions may propagate toward sensory cortices^61^^,^^62^ through beta-band (13–20 Hz) power dynamics^63^^,^^64^ and delta-beta phase-amplitude coupling.^65^^,^^66^ Future work may shed further light on the extent to which the prefrontal cortex orchestrates syllable and chunk tracking in left and right fronto-temporal areas and whether statistical learning operates a predictive routing.^67^^,^^68^

One additional limitation of our study relates to the narrow time windows of our ERF effects. The narrow latencies of interest were extracted based on the FWHM of the M200 and M400 grand-averaged peaks. This procedure was adopted in order to approximate a fractional area latency around grand-averaged peaks, which is preferable to using only the peak latency,^69^^,^^70^ especially for analyzing difference waves.^71^ In line with prior studies,^29^^,^^30^^,^^31^^,^^32^ we tested differences across conditions within the FWHM latencies, as well as in the full *a priori* windows. These control analyses suggest a robust PR effect on the M200 but only a marginal TP effect on the M400, which should thus be interpreted with caution. Furthermore, our behavioral analyses did not show a significant effect of learning in the PR– TP + condition, which might be due to the low reliability of the 2-AFC task in assessing post-learning performance at the group level.^20^^,^^72^ Future studies may use a larger number of comparisons among items with varying levels of difficulty^72^ or different tasks^73^ to investigate individual differences in statistical learning.

## Resource availability

### Lead contact

Requests for further information and resources should be directed to and will be fulfilled by the lead contact, Lorenzo Titone (titone@cbs.mpg.de).

### Materials availability

This study did not generate new unique reagents.

### Data and code availability

• The original data reported in this study cannot be deposited in a public repository because of ethical restrictions. The aggregated data and summary statistics have been deposited at Zenodo and are publicly available as of the date of publication at: https://doi.org/10.5281/zenodo.17990155.
• Stimuli and analysis code have been deposited at OSF and is publicly available as of the date of publication at https://doi.org/10.17605/OSF.IO/QTRZ9.
• Any additional information required to reanalyze the data reported in this paper is available from the lead contact upon request.

## Acknowledgments

The study was supported by the 10.13039/501100004189Max Planck Society through the award of the independent MPRG Language Cycles to L.M. The authors thank Andrea E. Martin for comments on the study design; Caroline Duchow, Yvonne Wolff-Rosier, Heike Böthel, and David Weyer for support with data collection; and Jule Nabrotzky, Lena Henke, and Kerstin Flake for help with visualizations.

## Author contributions

Conceptualization, L.T. and L.M.; methodology, L.T. and B.M.; data curation, L.T. and B.M.; investigation, L.T. and B.M.; formal analysis, L.T.; validation, L.T. and LM.; resources, B.M.; visualization, L.T. and L.M.; project administration, L.T. and L.M.; supervision, L.M.; funding acquisition, L.M.; writing – original draft, L.T.; writing – review and editing, L.T., B.M., and L.M.

## Declaration of interests

The authors declare no competing interests.

## Declaration of generative AI and AI-assisted technologies in the writing process

During the preparation of this work, the authors did not use Generative AI.

## STAR★Methods

### Key resources table

**Table:** 

REAGENT or RESOURCE	SOURCE	IDENTIFIER
**Deposited data**		

Dataset for Neural tracking of prosodic and statistical rhythms jointly supports artificial language learning	This paper	https://doi.org/10.5281/zenodo.17990155
Code for Neural tracking of prosodic and statistical rhythms jointly supports artificial language learning	This paper	https://doi.org/10.17605/OSF.IO/QTRZ9

**Software and algorithms**		

ALPARC	Titone et al. (2026)	https://github.com/milosen/alparc
Psychtoolbox-3	Brainard (1997)	http://psychtoolbox.org
MATLAB (version R2024b)	Mathworks	https://www.mathworks.com/products/matlab.html
Fieldtrip (version 0.20250420)	Oostenveld et al. (2011)	https://www.fieldtriptoolbox.org/download/
MaxFilterTM (version 2.2.15)	Elekta Neuromag, Helsinki, FI.	https://www.elekta.com/
FreeSurfer	The General Hospital Corporation, Boston, MA, USA.	http://surfer.nmr.mgh.harvard.edu/

**Other**		

Human participants	This paper	N/A

### Experimental model and study participant details

#### Participants

32 German native speakers (16 females, 16 males, mean age = 29 ± 4, age range: 22–36) participated in the study. We did not assess the influence of sex or gender on the results of our study. All participants were right-handed, had normal hearing, normal or corrected-to-normal vision, and reported no prior history of neurological, learning, speech, or language disorders. Participants were assigned chronologically to one of the eight lists counterbalancing the order of presentation of the four experimental conditions and the artificial lexicon that was used in the prosody contrast (Table S1). The experiment conformed to the guidelines of the Declaration of Helsinki and was approved by the local ethical committee of the University of Leipzig, DE (ref. 076/22ek). Participants were naive to the purpose of the study, provided their informed consent prior to the experiment, and were reimbursed thereafter.

### Method details

#### Stimuli

We generated two artificial languages of four trisyllabic pseudowords using a custom Python toolbox that controls phonological, acoustic, statistical, and linguistic confounds in artificial language learning paradigms.^24^ For the current study, consonant-vowel syllables were pooled from a German corpus,^74^ and filtered to retain only syllables with long vowels in order to uniformize phoneme timing. In addition, we excluded syllables whose frequency of use deviated from a uniform distribution (α = 0.05, two-sided) to ensure that artificial language learning would not be biased by too (in)frequent syllables.

To control for phonotactics, we extracted the manner and place of articulation for each consonant^75^ based on a binary matrix of phonological features for each phoneme. Syllables were thus combined into triplets to shape pseudowords that obey the obligatory contour principle,^76^ a rule that was reported to partially account for pseudoword tracking.^21^ To control that, our custom algorithm ensured that the manner and place of articulation and vowel identity were different across all three syllables within each pseudoword. Next, we excluded pseudowords that two native German speakers rated as extremely (dis)similar to real German words. We then used phonemic bigram, trigram, and positional probabilities from another German corpus^77^ to filter out pseudowords that contained too (in)frequent phoneme bigrams (α = 0.05, two-sided), or trigram or word onset phonemes that were not in the corpus. Based on the resulting inventory of pseudowords, we generated lexicons of four trisyllabic pseudowords with the lowest possible amount of phonological feature repetitions among phonemes that occupy the same ordinal position across pseudowords. This way, we could minimize the phonological rhythmicity in the syllable streams, which would otherwise be confounded with the TP patterns at the pseudoword rate.^24^

For each lexicon of four pseudowords (with a general *ABC* syllable structure), we also built sets of four part-words (with a general *C’AB* syllable structure), by using all 12 unique syllables in the lexicon. We then retained only those lexicons for which at least one full set of four part-words violated at least one phonotactic constraint, and for which at least twelve pairs of pseudowords and part-words had maximum phonemic Levenshtein distance and also covered the same number of repetitions of pseudoword and part-words. Thus, each pseudoword was paired against three distinct part-words on separate trials, always leaving a different part-word out across the different pseudowords being tested. For each lexicon that fulfilled these criteria, we thus obtained 12 pairs of pseudowords and part-words with constant Levenshtein distance, but opposite adherence to our phonotactic rule. Finally, we selected two distinct lexicons of pseudowords (L1: *ho-bi-schä*, *nü-gi-fa*, *po-sa-hö*, *fo-hi-tu*; L2: *ka-fu-ri*, *mü-ko-zu*, *schö-he-pi*, *hö-de-va*) and corresponding part-words (L1’: *hö-nü-gi*, *fa-ho-bi*, *tu-po-sa*, *schä-fo-hi*; L2’: *pi-ka-fu*; *ri-mü-ko*; *va-schö-he*; *zu-hö-de*) with the smallest overlap of syllables and the largest phonemic Levenshtein distance across all elements. Based on our two artificial lexicons, we generated different types of syllable streams, by employing a custom approach that ensures TP stationarity throughout the streams.^24^ In TP + streams, pseudowords followed one another uniformly, leading to stable TPs of 1 and 0.33 within and across pseudoword boundaries, respectively. In contrast, in TP– streams, the same syllables always occupy the same position in a chunk by repeating at multiples of three, but each transitioned uniformly to any syllable in the next position of any pseudoword, leading to stationary syllable TPs of 0.25 throughout the whole stream. This constraint equates acoustic spectral differences between TP+ and TP– streams at the pseudoword rate.^20^

To manipulate prosody, we extracted the pitch of a familiar German intonation unit. We employed Wavenet^78^ and speech synthesis markup language tags^79^ to attain a correct pronunciation was assessed by a native speaker. The contour automatically produced by the speech synthesizer showed a strong-weak-weak prosodic pattern, resolving an attachment ambiguity of the relative clause in the sentence: “*Bo kennt Sue, die sehr stinkt* (*Bo knows Sue, who stinks*)”, by prosodically marking the subject of the main clause (underlined). We edited the audio output using custom Praat scripts,^80^ mimicking the editing of individual syllables. The first three words of the prosodically-marked sentence were isolated, trimmed, smoothed, and stretched to a total duration of 300 ms. From the three concatenated segments, we extracted the 900-ms-long pitch contour to replace the pitch tier of all consecutive chunks in PR + streams.

All of the syllables composing our two artificial lexicons were synthesized with Wavenet^78^ with occasional manual editing of synthesis markup language tags^79^ to attain a correct pronunciation, which was assessed by a native speaker. The audio files were then edited in Praat.^80^ Silences before and after the onset and offset of a syllable were removed until the sound amplitude exceeded a threshold of 0.01; the 10-ms edges on both ends were trimmed and smoothed; the duration of each syllable was stretched or compressed to a fixed duration of 280 ms and enclosed by 10 ms of silence on both ends, leading to a total duration of 300 ms per audio file. Finally, the sound amplitude was normalized to 65 dB. Syllables were concatenated to compose continuous auditory streams, according to the pseudorandomized structure of TP to be used in the exposure phase, and according the trisyllabic sequences that shaped pseudowords and part-words to be used in the recognition phase.

#### Procedure

Participants were presented with four conditions (PR– TP–; PR– TP+; PR + TP–; and PR + TP+) in a within-subject design. Each condition encompassed one exposure phase and one recognition phase. The order of presentation of these conditions followed a balanced Latin square design, so that each condition preceded and followed every other condition uniformly across participants. The assignment of either of the two artificial languages to the PR + or to the PR– condition was counterbalanced across participants. Self-paced breaks split each phase into two mini blocks of about 3 min each. A longer break was given at the end of each exposure or recognition phase, while the MEG recording was restarted. In the exposure phase, participants listened to the syllable streams (576 syllables = 192 triplets = 172.8 s per mini block) in one of the four conditions. In PR + streams, a natural pitch modulation consistently marked trisyllabic contours by a strong-weak-weak acoustic pattern. In PR− streams, all syllables kept their original pitch. In parallel, syllables were arranged in a TP-structured stream (TP+), with syllable TPs of 1 and 0.33 within and across pseudoword boundaries, or separately scrambled into a TP-uniform stream (TP−) with fixed syllable TPs of 0.25 throughout the whole stream. After each exposure phase (∼6 min total per condition), participants performed a two-alternative forced-choice recognition task. In each trial (*n* = 48 per condition), two trisyllabic sequences (*ABC* pseudowords vs. *C’AB* part-words) were presented sequentially and participants had to choose one as more familiar. Overall, twelve unique pairs of pseudowords and part-words (Table S2) were presented twice per mini block with counterbalanced order of the two test chunks. This task was presented twice per condition (i.e., two consecutive mini blocks embedded in the recognition phase) for a total of 48 trials. Moreover, the same recognition phase was presented after both TP+ and TP- conditions using the same lexicon, leading to the same set of items being tested twice per lexicon. As a result, we coded the first exposure to the task as HI–, and the second exposure to the same lexicon as HI+. In each trial, the two test chunks were played sequentially with a jittered inter-trial interval of 1.5–2 s, and a jittered inter-stimulus interval of 1–1.5 s. One second after the offset of the second chunk, participants had to respond which of the two chunks was more familiar based on the prior exposure phase through a button press with the left or right hand. Importantly, all test chunks had no prosody manipulation and the same set of stimuli was used in either TP condition.

### Quantification and statistical analysis

#### Data acquisition

The experiment was conducted in an electromagnetically shielded room in a single session. Stimuli were presented using Psychtoolbox-3.^81^ MEG-compatible air-conduction earplugs (ER3-14 A/B, Etymotic Research Inc., Elk Grove Village IL, USA) connected via a 50-cm plastic tube to piezo-phones (TIP-300, Nicolet, Biomedical Madison, WI, USA) were used to transmit the auditory stimuli. A Panasonic PT-D7700E (Matsushita Electric Industrial Co., Japan) exterior to the shielded room was used to present the visual instructions on a semi-transparent screen located ∼90 cm away from the participants, who sat inside a 306-channel MEG system (Vectorview Elekta-Neuromag, Helsinki, Finland). The head position inside the MEG helmet was monitored with five head-tracking coils. MEG signals were recorded at a sampling rate of 1,000 Hz and online band-pass filtered at 0–330 Hz. Moreover, the vertical and horizontal electrooculogram (EOG) and the electrocardiogram were also recorded with three sets of bipolar electrodes, respectively placed above and below the left eye; on the outer canthi of both eyes; and on the right shoulder plate and left rib. The ground electrode was placed on the left mastoid bone. Participants’ head shape was digitized with a Polhemus FASTRAK 3D digitizer before the MEG recording. For source reconstruction, individual T1-weighted magnetic resonance imaging (MRI) volumes were previously acquired with a 3T scanner (Magnetom Trio, Siemens AG, Germany).

#### Data preprocessing

Raw MEG data were browsed in MNE-Python for visual selection of noisy channels. External noise and head motion were corrected with MaxFilter™ software (Version 2.2.15, Elekta Oy, FI). The head position was realigned to the position in the block that was the closest to the overall mean of the head-tracking coils. Visually marked noisy channels were interpolated by block. Spherical models were created with the Signal Source Separation (SSS) method^82^ up to the 11^th^ order for the internal head field model and 2^nd^ order for the environmental field model. Head movements were compensated using a 500-ms window while forwarding with steps of 25 ms. The temporal suppression extension to the SSS method was used to project out correlations higher than 0.95 between the internal and external field components using a 20-s window.

After external noise and motion correction using MaxFilter™, we further processed the MEG data with MATLAB (version R2024b, MathWorks Inc.) using the FieldTrip toolbox.^83^ The self-paced break in-between two mini blocks were cut out from the data. The resulting two continuous MEG segments per block were detrended, demeaned, and band-passed filtered between 0.1 Hz and 30 Hz (one-pass zero-phase shift finite impulse response filter windowed sync, Kaiser window type). We then transformed the data from an original 306-sensor-space (with 102 magnetometers, 204 gradiometers) to a reconstructed 510-channel-space with a fixed magnetometer-like scale, by disentangling the positive and the negative component of each gradiometer. In the exposure blocks, we segmented the continuous data into 900-ms-long non-overlapping epochs including consecutive chunks of three syllables. In the recognition blocks, the MEG data were time-locked to stimulus onsets and segmented into 1.5-*s*-long epochs, including a baseline of 250 ms.

To identify common-mode artifacts (e.g., SQUID jumps; sensors heating), we used an automatic artifact detection algorithm that finds abrupt changes in the signal at all channels (z-cutoff = 20). Likewise, epochs containing blinks and saccades were marked automatically based on the EOG signals (z-value cutoff = 4, bandpass filter = 2–15 Hz). Epochs containing common-mode artifacts were provisionally removed to improve the signal-to-noise prior to an independent component analysis (ICA) on 20 principal components. ICs were inspected along with the MEG and EOG epochs marked for eye artifacts. ICs that captured eye blinks, saccades, and heartbeats were excluded from back-projection to the MEG data (removed ICs: mean = 2.95; std = 0.76; range = 1–7). Residual artifacts after the ICA correction were identified automatically (z-cutoff = 50) in order to inform a visual inspection before rejection (rejected trials: exposure: mean = 0.5%, std = 0.5%; recognition: mean = 3.9%, std = 3.5%).

#### Frequency-domain analyses

To analyze the power spectral density of the MEG signal in the exposure phase, we first concatenated consecutive artifact-free epochs within each block and padded them beyond the maximum trial duration (i.e., up to 180 s) to uniformize the frequency resolution. Next, we computed the Fast Fourier Transform using Hann tapers (0.2–4 Hz, resolution 0.01 Hz), averaging across the concatenated artifact-free segments. To assess the significance of the spectral peaks within each condition, the observed power at each individual frequency was compared to the average power across the corresponding neighboring frequencies (i.e., the baseline power) by performing separate cluster-based permutation tests,^25^ specifying a paired samples *t* statistic based on 1,000 montecarlo randomizations, a minimum three channels per cluster, and a one-sided corrected alpha threshold of 0.05. Then, the normalized power was computed as the ratio of the observed power and the baseline power.

Based on our spectral density results, we could identify two frequencies of interest for later analyses, that is, the syllable rate (3.33 Hz) and the chunk rate (1.11 Hz). We thus used a wavelet method to compute the Fourier spectrograms at these two frequencies of interest based on the continuous data. We then reshaped the Fourier spectrograms into 0.9-*s*-long non-overlapping epochs to calculate ITPC at both rates across artifact-free trials. To assess ITPC differences at the two rates of interest based on the presence (or absence) of PR or TP patterns, we modeled the effects of our factors of interest (PR and TP) on ITPC as a 2 × 2 ANOVA. We modeled the main effects of PR and TP (at each rate) as the average ITPC difference between PR+ and PR− streams and between TP+ and TP− streams, respectively. In addition, we modeled the PR × TP interaction as the difference of ITPC differences, as follows: [(TP + PR+) – (TP + PR-)] – [(TP- PR+) – (TP- PR-)]. For each contrast, we assessed the unidirectional hypotheses that ITPC at the chunk rate (1.11 Hz) increased, while ITPC at the syllable rate (3.33 Hz) decreased, by performing separate cluster-based permutation tests,^25^ for which we specified a paired samples *t* statistic based on 10,000 montecarlo randomizations, a minimum of three channels per cluster, and a one-sided corrected alpha threshold of 0.05. Furthermore, we assessed the temporal evolution of ITPC throughout each exposure stream by calculating a simple moving average of ITPC over a 45-*s*-long window (i.e., spanning 50 chunks of three syllables). We then aimed to test the fixed effect of time on ITPC in the respective sensor-level cluster that showed an effect at the chunk rate due to presence of PR or TP pattern. To that end, we designed separate mixed-effects models^28^ with a random intercept per participant for the two-halves of exposure in each condition.

#### Time-domain analyses

In the recognition phase, we analyzed the differences in the M200 and M400 event-related field (ERF) responses to pseudowords and part-words. First, to identify the peak latencies of interest in our dataset, we computed the root-mean-square (RMS) of the grand-average MEG signal across all trials, sensors, and participants. We searched for peaks in the rms within temporal windows (170–250 ms and 300–500 ms) informed by the prior literature on the P200 and N400 event-related potentials.^16^ We thus identified the latencies of interest in our dataset as the full-width-half-max (FWHM) of the RMS peaks in each temporal window, that is, 196–242 ms and 435–470 ms, respectively. For control analyses, we averaged the ERFs in the full *a priori* latency windows, or in a ±50 ms, range around the peak.

To calculate the PR and TP effects, we first split the test chunks by type (pseudowords and part-words), applied baseline correction (−150 ms and 0 ms relative to stimulus onset), and a high-pass filtered the epoched data (using a finite impulse response window sync filter with a 1 Hz cutoff) to remove residual low-frequency trends from the signal. We averaged the responses to pseudowords and part-words separately for each condition and computed the raw difference between part-words and pseudowords within each condition, sensor, and participant. To assess differences between part-words and pseudowords, after PR + versus PR− and after TP + versus TP− conditions at the two ERF latencies of interest, we modeled the PR × M200 effect as the M200 difference in PR + versus PR− conditions and the TP × M400 effect as the M400 difference in TP + versus TP− conditions. Likewise, we modeled the PR × M400 and TP × M200 effects. For each contrast, we performed a cluster-based permutation test^25^ averaging within the respective latency of interest, and specifying a paired samples *t* statistic based on 10,000 montecarlo randomizations, a minimum of three channels per cluster, and a one-sided corrected alpha threshold of 0.05. In one additional control analyses, we tested for ERF effects on the whole post-stimulus window of 1 s (i.e., without averaging over time in *a priori* windows), reducing the number of randomizations to 1,000.

#### Source analyses

For source reconstruction of the MEG signal, participants’ MRI brain scans were segmented using FreeSurfer (http://surfer.nmr.mgh.harvard.edu/) and co-registered to the MEG data by aligning the scalp points digitized with Polhemus^84^ using MNE-Python. Individualized head models were created using the single-shell method.^85^ Boundary element models with inner-skull surfaces with 2,562 vertices served as volume conductor models. Source models with 20,484 vertices were created with MNE-Python and parcellated into the 360 regions of the HCP-MMP1 atlas.^27^ Individual lead-fields were created for each participant and block, using normalization to reduce the depth bias. Forward modeling was successful for 25/32 participants due to lack of MRI scans (five) or co-registration failure (two).

Source reconstruction of the frequency-domain MEG signal from the exposure phase was based on dynamic imaging of coherent sources^26^ with using separate cross-spectral density matrices for the two rates of interest. Source-level Fourier spectrograms were thus obtained by multiplying each frequency-specific filter with the sensor-level spectrograms for each condition. We then reduced the tripartite moment orientations to one dimension using singular value decomposition and a root-mean-square . Next, we reshaped the source-level Fourier spectrograms in time to compute the ITPC across non-overlapping epochs spanning consecutive chunks of three syllables.

Source reconstruction of the time-domain MEG signal from the recognition phase was based on linear-constrained minimum-variance beamforming^33^ with a 5% lambda and a fixed orientation. For each block, we constructed a common filter encompassing all trials (i.e., with both pseudowords and part-words) based on the covariance matrix from the peri-stimulus window (i.e., −150 ms to 1,000 ms locked to stimulus onset). The source-level fields were reconstructed separately for each condition, by convolving the pre-computed common filter with the sensor-level traces time-locked to the presentation of pseudowords and part-words. We then averaged the signal from all source-level parcels within each of the 360 atlas regions and applied the same operations as in the sensor-level time-domain analyses to reconstruct the significant interactions of PR or TP and the ERF at the source level. (i.e., subtracting responses to pseudowords from part-words, averaging within PR and TP factor levels and within the M200 and M400 latencies of interest).

#### Behavioral analyses

Our behavioral analyses focused on participants’ accuracy in recognizing pseudowords as more familiar than part-words. To that end, we first computed the average accuracy for all participants within each condition and tested it against chance-level (50%) using separate one-sample t-tests. We then aimed to test the effects of PR and TP on recognition accuracy using linear mixed-effect models.^28^ At this stage, we also considered potential carry-over effects of experimental history (HI) due to the within-subject nature of our study design. In fact, the same set of test stimuli for the recognition phase was used after both TP+ and TP− streams. Additionally, the order of presentation of the experimental conditions varied across participants based on a balanced Latin square design.

Based on our study design, the history in which conditions were presented could bias task performance. In fact, pseudowords and part-words may have occasionally been known to the participant based on a prior recognition phase, regardless of the PR and TP patterns in the immediately preceding exposure phase. We thus aimed to test whether knowing the pseudowords from a previous TP + condition would bias the responses in a subsequent TP− condition that presented the same set of stimuli. To that end, we compared a binomial linear mixed-effects model with only a random intercept per participant against an extended mixed-effects model that added the fixed factors of PR, TP, and HI, and their interactions.

### Additional resources

Additional resources for stimuli validation metrics can be found at: https://github.com/milosen/alparc.

## Supplemental information

Document S1. Figures S1 and S2, and Tables S1–S4

Data S1. Auditory stimuli for each lexicon (L), prosody (yes/no P), TP (struct/random) and trial (T)
