# Supplementary material for: Neural tracking of prosodic and statistical rhythms jointly supports artificial language learning
Source: iScience. 2026 Jun 24;29(7):116458. doi: 10.1016/j.isci.2026.116458 (PMC13320337; doi:10.1016/j.isci.2026.116458)
Supplement: Document S1. Figures S1 and S2, and Tables S1–S4 [file mmc1.pdf]

iScience, Volume *29*

Supplemental information

Neural tracking of prosodic and statistical rhythms jointly supports artificial language learning

Lorenzo Titone, Burkhard Maess, and Lars Meyer

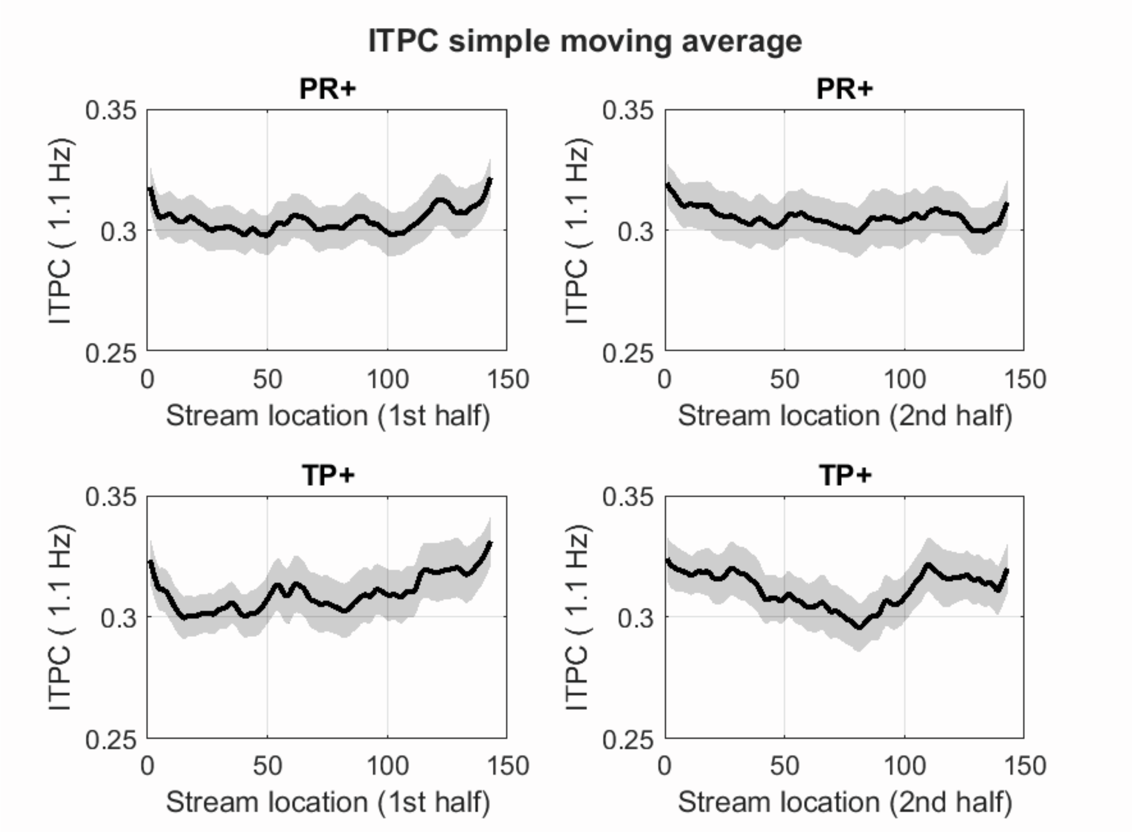

Figure S1. Moving average of inter-trial phase coherence in the 1.11 Hz clusters as a function of time. The panels display the time course of ITPC across stream locations computed using a simple moving average over 50 overlapping epochs, each including one pseudoword. The left and right panels consider the first and second halves of the exposure phase separately. The top and bottom panels display the grand-averaged time course of ITPC across subjects, within the cluster of sensors that displayed significant differences in the PR contrast (Figure 3A), or in the TP contrast (Figure 3B), respectively. Shaded areas represent the SEM.

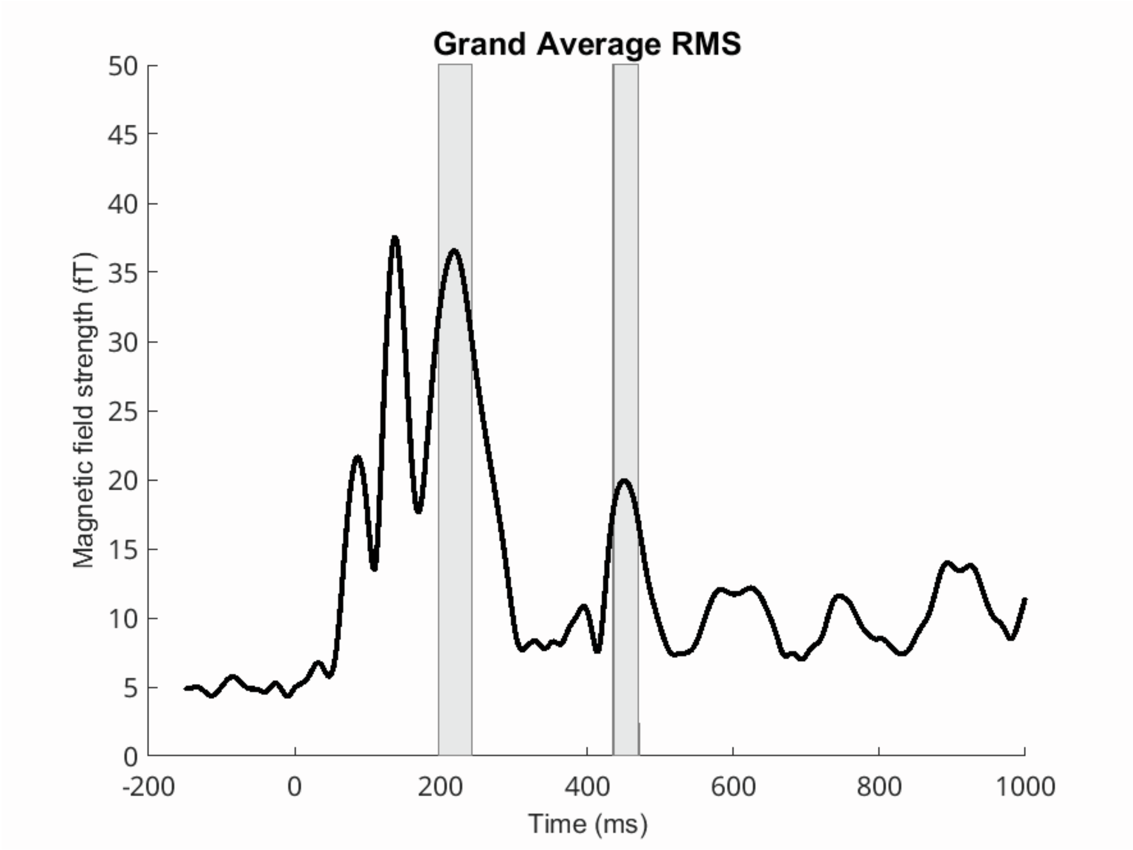

Figure S2. Grand-averaged root-mean-square signal across all test trials in the recognition phase. Grey areas represent the full-width-half-max (FWHM) of the RMS peaks identified within two windows of interest that correspond to the M200 (170-250 ms) and M400 (350-500 ms) latencies. The FWHM was computed as the full width at half the maximum magnitude (relative to the mean of the minimum magnitude at the left and right-hand sides of the peak) within each time window. This procedure resulted in more restricted windows of interest for the M200 and M400, namely: 196-242 ms (Figure 4A), and 435-470 ms (Figure 4B), respectively.

Table S1

Types of list counterbalancing the order of presentation of each condition and the lexicon used for PR+

List ID	Lexicon ID	Condition 1	Condition 2	Condition 3	Condition 3
1	1	L2 PR– TP– HI–	L2 PR– TP+ HI+	L1 PR+ TP+ HI–	L1 PR+ TP– HI+
2	2	L1 PR– TP– HI–	L1 PR– TP+ HI+	L2 PR+ TP+ HI–	L2 PR+ TP– HI+
3	1	L2 PR– TP+ HI–	L1 PR+ TP– HI–	L2 PR– TP– HI+	L1 PR+ TP+ HI+
4	2	L1 PR– TP+ HI–	L2 PR+ TP– HI–	L1 PR– TP– HI+	L2 PR+ TP+ HI+
5	1	L1 PR+ TP– HI–	L1 PR+ TP+ HI+	L2 PR– TP+ HI–	L2 PR– TP– HI+
6	2	L2 PR+ TP– HI–	L2 PR+ TP+ HI+	L1 PR– TP+ HI–	L1 PR– TP– HI+
7	1	L1 PR+ TP+ HI–	L2 PR– TP– HI–	L1 PR+ TP– HI+	L2 PR– TP+ HI+
8	2	L2 PR+ TP+ HI–	L1 PR– TP– HI–	L2 PR+ TP– HI+	L1 PR– TP+ HI+

Table S2
Test trials in the recognition phase

Lexicon	Trial ID	Pseudoword	Part-word
1	1	*po-sa-hö*	*hö-nü-gi*
1	2	*po-sa-hö*	*fa-ho-bi*
1	3	*po-sa-hö*	*tu-po-sa*
1	4	*nü-gi-fa*	*hö-nü-gi*
1	5	*nü-gi-fa*	*fa-ho-bi*
1	6	*nü-gi-fa*	*schä-fo-hi*
1	7	*ho-bi-schä*	*fa-ho-bi*
1	8	*ho-bi-schä*	*tu-po-sa*
1	9	*ho-bi-schä*	*schä-fo-hi*
1	10	*fo-hi-tu*	*hö-nü-gi*
1	11	*fo-hi-tu*	*tu-po-sa*
1	12	*fo-hi-tu*	*schä-fo-hi*
2	1	*schö-he-pi*	*pi-ka-fu*
2	2	*schö-he-pi*	*ri-mü-ko*
2	3	*schö-he-pi*	*va-schö-he*
2	4	*ka-fu-ri*	*pi-ka-fu*
2	5	*ka-fu-ri*	*ri-mü-ko*
2	6	*ka-fu-ri*	*zu-hö-de*
2	7	*hö-de-va*	*pi-ka-fu*
2	8	*hö-de-va*	*va-schö-he*
2	9	*hö-de-va*	*zu-hö-de*
2	10	*mü-ko-zu*	*ri-mü-ko*
2	11	*mü-ko-zu*	*va-schö-he*
2	12	*mü-ko-zu*	*zu-hö-de*

Table S3

Paired-sample t-test results for the ITPC contrasts in all regions with p < .05 (uncorrected)

Contrast	Atlas Region	t	df	p-value	FDR-p	CI 95%
**PR × 1.1 Hz**	L_PFop	3.55	24	0.00082	0.106	[5.393E-04 Inf]
PR × 1.1 Hz	L_LO2	3.49	24	0.00094	0.106	[8.179E-04 Inf]
PR × 1.1 Hz	L_VVC	3.41	24	0.00116	0.106	[6.473E-04 Inf]
PR × 1.1 Hz	L_V8	3.40	24	0.00118	0.106	[8.725E-04 Inf]
PR × 1.1 Hz	L_LO1	3.24	24	0.00174	0.116	[5.208E-04 Inf]
PR × 1.1 Hz	L_a9-46v	3.11	24	0.00239	0.116	[4.472E-04 Inf]
PR × 1.1 Hz	L_V4t	3.06	24	0.00269	0.116	[4.706E-04 Inf]
PR × 1.1 Hz	L_OP1	2.96	24	0.00345	0.116	[4.700E-04 Inf]
PR × 1.1 Hz	L_9-46d	2.95	24	0.00348	0.116	[4.466E-04 Inf]
PR × 1.1 Hz	L_A1	2.94	24	0.00359	0.116	[4.106E-04 Inf]
PR × 1.1 Hz	L_p10p	2.90	24	0.00393	0.116	[3.763E-04 Inf]
PR × 1.1 Hz	L_V4	2.86	24	0.00428	0.116	[4.423E-04 Inf]
PR × 1.1 Hz	L_PIT	2.83	24	0.00460	0.116	[6.334E-04 Inf]
PR × 1.1 Hz	L_TE2p	2.82	24	0.00479	0.116	[5.534E-04 Inf]
PR × 1.1 Hz	L_FFC	2.81	24	0.00483	0.116	[5.546E-04 Inf]
PR × 1.1 Hz	L_VMV3	2.74	24	0.00566	0.127	[4.477E-04 Inf]
PR × 1.1 Hz	L_9a	2.66	24	0.00692	0.147	[3.723E-04 Inf]
PR × 1.1 Hz	L_PGp	2.61	24	0.00765	0.148	[2.476E-04 Inf]
PR × 1.1 Hz	L_MBelt	2.60	24	0.00788	0.148	[2.341E-04 Inf]
PR × 1.1 Hz	L_PFcm	2.58	24	0.00825	0.148	[2.281E-04 Inf]
PR × 1.1 Hz	L_AIP	2.51	24	0.00969	0.166	[2.830E-04 Inf]
PR × 1.1 Hz	L_TE1m	2.48	24	0.01020	0.167	[3.738E-04 Inf]
PR × 1.1 Hz	L_TE2a	2.44	24	0.01134	0.171	[2.247E-04 Inf]
PR × 1.1 Hz	L_p32	2.43	24	0.01140	0.171	[2.434E-04 Inf]
PR × 1.1 Hz	L_TE1p	2.37	24	0.01313	0.189	[2.962E-04 Inf]
PR × 1.1 Hz	L_TF	2.33	24	0.01438	0.195	[1.766E-04 Inf]
PR × 1.1 Hz	L_10d	2.32	24	0.01465	0.195	[2.057E-04 Inf]
PR × 1.1 Hz	L_STSdp	2.30	24	0.01520	0.195	[1.850E-04 Inf]
PR × 1.1 Hz	L_p32pr	2.27	24	0.01639	0.200	[2.891E-04 Inf]
PR × 1.1 Hz	L_V3B	2.26	24	0.01663	0.200	[1.617E-04 Inf]
PR × 1.1 Hz	L_PH	2.20	24	0.01871	0.212	[2.577E-04 Inf]
PR × 1.1 Hz	L_PFt	2.19	24	0.01918	0.212	[1.947E-04 Inf]
PR × 1.1 Hz	L_V7	2.18	24	0.01948	0.212	[1.524E-04 Inf]

Contrast	Atlas Region	t	df	p-value	FDR-p	CI 95%
PR × 1.1 Hz	L_A5	2.16	24	0.02040	0.212	[1.297E-04 Inf]
PR × 1.1 Hz	L_DVT	2.15	24	0.02090	0.212	[1.571E-04 Inf]
PR × 1.1 Hz	L_V3CD	2.14	24	0.02119	0.212	[1.458E-04 Inf]
PR × 1.1 Hz	L_PHT	2.11	24	0.02290	0.219	[1.521E-04 Inf]
PR × 1.1 Hz	L_PHA3	2.09	24	0.02352	0.219	[1.460E-04 Inf]
PR × 1.1 Hz	L_VMV2	2.08	24	0.02438	0.219	[1.270E-04 Inf]
PR × 1.1 Hz	L_a24	2.04	24	0.02620	0.219	[1.077E-04 Inf]
PR × 1.1 Hz	L_V6A	2.03	24	0.02686	0.219	[1.691E-04 Inf]
PR × 1.1 Hz	L_OP2-3	2.02	24	0.02752	0.219	[1.499E-04 Inf]
PR × 1.1 Hz	L_STSvp	2.01	24	0.02769	0.219	[1.382E-04 Inf]
PR × 1.1 Hz	L_A4	2.01	24	0.02806	0.219	[8.200E-05 Inf]
PR × 1.1 Hz	L_8Ad	1.98	24	0.02944	0.219	[1.210E-04 Inf]
PR × 1.1 Hz	L_46	1.98	24	0.02957	0.219	[1.102E-04 Inf]
PR × 1.1 Hz	R_a10p	1.98	24	0.02962	0.219	[9.901E-05 Inf]
PR × 1.1 Hz	L_H	1.97	24	0.03049	0.219	[6.801E-05 Inf]
PR × 1.1 Hz	L_PBelt	1.97	24	0.03053	0.219	[7.122E-05 Inf]
PR × 1.1 Hz	L_9p	1.96	24	0.03063	0.219	[1.360E-04 Inf]
PR × 1.1 Hz	L_LBelt	1.96	24	0.03108	0.219	[6.680E-05 Inf]
PR × 1.1 Hz	L_a24pr	1.94	24	0.03228	0.223	[1.316E-04 Inf]
PR × 1.1 Hz	L_9m	1.93	24	0.03282	0.223	[6.073E-05 Inf]
PR × 1.1 Hz	L_OP4	1.88	24	0.03606	0.235	[6.593E-05 Inf]
PR × 1.1 Hz	L_p47r	1.88	24	0.03609	0.235	[6.296E-05 Inf]
PR × 1.1 Hz	L_a32pr	1.87	24	0.03665	0.235	[9.013E-05 Inf]
PR × 1.1 Hz	L_FOP1	1.87	24	0.03717	0.235	[5.604E-05 Inf]
PR × 1.1 Hz	L_Ig	1.85	24	0.03865	0.240	[6.907E-05 Inf]
PR × 1.1 Hz	L_IP2	1.78	24	0.04398	0.262	[2.406E-05 Inf]
PR × 1.1 Hz	L_2	1.77	24	0.04483	0.262	[2.463E-05 Inf]
PR × 1.1 Hz	L_PreS	1.77	24	0.04499	0.262	[1.687E-05 Inf]
PR × 1.1 Hz	L_IPS1	1.77	24	0.04506	0.262	[2.223E-05 Inf]
PR × 1.1 Hz	L_10v	1.75	24	0.04654	0.266	[1.270E-05 Inf]
PR × 1.1 Hz	L_1	1.74	24	0.04762	0.268	[1.025E-05 Inf]
PR × 1.1 Hz	L_V3	1.73	24	0.04869	0.270	[5.179E-06 Inf]
PR × 1.1 Hz	L_AVI	1.72	24	0.04954	0.270	[1.696E-06 Inf]
**TP × 1.1 Hz**	L_PHT	1.78	24	0.04382	0.973	[2.998E-05 Inf]

Contrast	Atlas Region	t	df	p-value	FDR-p	CI 95%
TP × 1.1 Hz	L_PH	1.77	24	0.04451	0.973	[2.917E-05 Inf]
TP × 1.1 Hz	L_TE1p	1.75	24	0.04637	0.973	[1.631E-05 Inf]
**PR × 3.3 Hz**	R_STSva	-2.34	24	0.01383	0.953	[-Inf -9.398E-05]
PR × 3.3 Hz	L_PFm	-2.30	24	0.01513	0.953	[-Inf -6.368E-05]
PR × 3.3 Hz	R_TE2a	-2.15	24	0.02085	0.953	[-Inf -6.108E-05]
PR × 3.3 Hz	R_TE1a	-2.10	24	0.02314	0.953	[-Inf -5.255E-05]
PR × 3.3 Hz	L_6v	-2.08	24	0.02397	0.953	[-Inf -5.145E-05]
PR × 3.3 Hz	R_TPOJ1	-2.04	24	0.02643	0.953	[-Inf -4.197E-05]
PR × 3.3 Hz	L_TE1m	-2.00	24	0.02861	0.953	[-Inf -3.977E-05]
PR × 3.3 Hz	R_TF	-1.97	24	0.02998	0.953	[-Inf -3.660E-05]
PR × 3.3 Hz	L_4	-1.91	24	0.03438	0.953	[-Inf -2.084E-05]
PR × 3.3 Hz	L_A1	-1.90	24	0.03483	0.953	[-Inf -2.668E-05]
PR × 3.3 Hz	R_TE1m	-1.87	24	0.03687	0.953	[-Inf -2.429E-05]
PR × 3.3 Hz	R_STGa	-1.77	24	0.04459	0.953	[-Inf -7.882E-06]
PR × 3.3 Hz	L_RI	-1.74	24	0.04765	0.953	[-Inf -3.234E-06]
**PR × TP × 3.3 Hz**	L_46	-2.53	24	0.00918	0.989	[-Inf -2.136E-04]
PR × TP × 3.3 Hz	L_31pd	-2.36	24	0.01335	0.989	[-Inf -1.616E-04]
PR × TP × 3.3 Hz	L_10pp	-2.17	24	0.02002	0.989	[-Inf -1.236E-04]
PR × TP × 3.3 Hz	L_10r	-2.04	24	0.02644	0.989	[-Inf -8.085E-05]
PR × TP × 3.3 Hz	L_8Ad	-2.03	24	0.02677	0.989	[-Inf -8.652E-05]
PR × TP × 3.3 Hz	L_31pv	-1.99	24	0.02876	0.989	[-Inf -7.212E-05]
PR × TP × 3.3 Hz	L_10d	-1.77	24	0.04482	0.989	[-Inf -1.213E-05]
PR × TP × 3.3 Hz	L_10v	-1.75	24	0.04612	0.989	[-Inf -9.660E-06]
PR × TP × 3.3 Hz	L_SFL	-1.72	24	0.04949	0.989	[-Inf -1.811E-06]

Table S4

Paired-sample t-test results for the ERF contrasts in all regions with p < .05 (uncorrected)

Contrast	Atlas Region	t	df	p-value	FDR-p	CI 95%
PR × M200	R_47s	3.00	24	0.00313	0.343	[4.892E-14 Inf]
PR × M200	R_pOFC	2.98	24	0.00323	0.343	[7.936E-14 Inf]
PR × M200	L_TE1a	2.95	24	0.00352	0.343	[3.178E-14 Inf]
PR × M200	R_25	2.86	24	0.00429	0.343	[1.006E-13 Inf]
PR × M200	R_s32	2.77	24	0.00533	0.343	[6.106E-14 Inf]
PR × M200	R_OFC	2.74	24	0.00573	0.343	[4.732E-14 Inf]
PR × M200	R_47m	2.50	24	0.00986	0.436	[3.385E-14 Inf]
PR × M200	L_H	2.48	24	0.01030	0.436	[3.628E-14 Inf]
PR × M200	R_13l	2.42	24	0.01163	0.436	[3.582E-14 Inf]
PR × M200	L_PHA1	2.31	24	0.01482	0.436	[3.664E-14 Inf]
PR × M200	L_TE1p	2.31	24	0.01499	0.436	[1.637E-14 Inf]
PR × M200	L_STSvp	2.21	24	0.01827	0.436	[2.293E-14 Inf]
PR × M200	R_a24	2.19	24	0.01933	0.436	[2.568E-14 Inf]
PR × M200	L_PreS	2.18	24	0.01958	0.436	[2.220E-14 Inf]
PR × M200	L_STSva	2.16	24	0.02066	0.436	[1.755E-14 Inf]
PR × M200	R_AAIC	2.15	24	0.02080	0.436	[1.516E-14 Inf]
PR × M200	R_10v	2.14	24	0.02141	0.436	[1.324E-14 Inf]
PR × M200	R_47l	2.12	24	0.02242	0.436	[1.114E-14 Inf]
PR × M200	R_p32	2.07	24	0.02446	0.436	[1.356E-14 Inf]
PR × M200	R_45	2.07	24	0.02481	0.436	[4.733E-15 Inf]
PR × M200	L_EC	2.02	24	0.02720	0.436	[1.773E-14 Inf]
PR × M200	R_11l	2.02	24	0.02732	0.436	[1.078E-14 Inf]
PR × M200	L_PI	2.01	24	0.02790	0.436	[1.433E-14 Inf]
PR × M200	L_TF	1.97	24	0.03034	0.455	[9.791E-15 Inf]
PR × M200	L_PHA2	1.92	24	0.03367	0.474	[1.151E-14 Inf]
PR × M200	L_s32	1.91	24	0.03425	0.474	[1.219E-14 Inf]
PR × M200	L_TE1m	1.88	24	0.03625	0.477	[6.482E-15 Inf]
PR × M200	R_MBelt	1.87	24	0.03711	0.477	[1.100E-14 Inf]
PR × M200	L_25	1.84	24	0.03897	0.477	[1.175E-14 Inf]

Contrast	Atlas Region	t	df	p-value	FDR-p	CI 95%
PR × M200	L_PeEc	1.82	24	0.04052	0.477	[4.889E-15 Inf]
PR × M200	R_10r	1.80	24	0.04209	0.477	[3.270E-15 Inf]
PR × M200	R_FOP2	1.80	24	0.04245	0.477	[5.408E-15 Inf]
PR × M200	R_FOP1	1.75	24	0.04633	0.505	[2.662E-15 Inf]
TP × M400	L_TGd	4.21	24	0.00016	0.056	[3.615E-14 Inf]
TP × M400	L_a10p	3.32	24	0.00142	0.256	[1.848E-14 Inf]
TP × M400	R_PGi	2.79	24	0.00504	0.605	[2.316E-14 Inf]
TP × M400	R_STV	2.59	24	0.00808	0.727	[1.731E-14 Inf]
TP × M400	L_13l	2.27	24	0.01633	0.869	[1.397E-14 Inf]
TP × M400	L_p10p	2.25	24	0.01694	0.869	[8.161E-15 Inf]
TP × M400	L_p32	2.19	24	0.01934	0.869	[1.017E-14 Inf]
TP × M400	L_10r	2.18	24	0.01972	0.869	[1.172E-14 Inf]
TP × M400	L_TGv	2.12	24	0.02243	0.869	[1.228E-14 Inf]
TP × M400	L_10pp	2.06	24	0.02517	0.869	[5.569E-15 Inf]
TP × M400	R_PGp	1.98	24	0.02955	0.869	[5.918E-15 Inf]
TP × M400	L_d32	1.97	24	0.03021	0.869	[5.829E-15 Inf]
TP × M400	L_PHT	1.95	24	0.03176	0.869	[3.399E-15 Inf]
TP × M400	L_47s	1.85	24	0.03844	0.869	[2.963E-15 Inf]
TP × M400	R_STSdp	1.85	24	0.03849	0.869	[2.834E-15 Inf]
TP × M400	R_V6A	1.85	24	0.03862	0.869	[3.346E-15 Inf]
TP × M400	L_OFC	1.75	24	0.04604	0.975	[9.835E-16 Inf]
TP × M400	L_Pir	1.72	24	0.04882	0.976	[4.836E-16 Inf]
